# Monitoring respiratory mechanics as a training tool for manual ventilation

**DOI:** 10.3389/fped.2025.1638566

**Published:** 2025-10-16

**Authors:** Carlos Eduardo Baldo Carlomagno, Renata Suman Mascaretti, João Cesar Lyra, Romy Schmidt Brock Zacharias, Mauricio Magalhaes, Pedro Alexandre Federico Breuel, Celso Moura Rebello

**Affiliations:** ^1^ Hospital Israelita Albert Einstein, São Paulo, Brazil; ^2^ Universidade Estadual Paulista Julio de Mesquita Filho Faculdade de Medicina, Botucatu, Brazil; ^3^ Faculdade de Ciencias Medicas da Santa Casa de Sao Paulo, São Paulo, Brazil; ^4^ Hospital Municipal e Maternidade Escola de Vila Nova Cachoerinha Dr Mário de Moraes Altenfelder, São Paulo, Brazil

**Keywords:** respiratory function, manual ventilation, neonatal resuscitation, tidal volume (VT), mask leakage, training-coaching-monitoring, residency and fellowship

## Abstract

**Introduction:**

Approximately 500,000 newborns in Brazil require respiratory support at birth each year. Manual ventilation is essential in neonatal resuscitation, but achieving adequate tidal volume (Tv) delivery and minimizing face mask leakage remain challenging. Respiratory Function Monitors (RFMs) offer real-time feedback that may enhance training effectiveness. This study aimed to assess the impact of RFM use on improving manual ventilation skills among neonatology residents using self-inflating bags (SIB) and T-piece resuscitators, focusing on optimizing Tv delivery and reducing mask leakage.

**Methods:**

A prospective experimental study was conducted with 23 neonatology residents from four training programs. Participants performed manual ventilation on a neonatal manikin across five sessions: baseline without RFM (V1), with RFM feedback (V2), immediately post-training without RFM (V3), and follow-ups at one month (V4) and three months (V5) without RFM. Tidal volume and mask leakage were recorded using a computerized acquisition system. Data were analyzed using mixed linear models for repeated measures.

**Results:**

The participants had a mean age of 29 years; 91.3% were female, and 60.9% were first-year residents. At baseline, SIB ventilation resulted in excessive Tv [mean: 10.43 ml/kg (95% CI: 9.15–11.72)]. Following RFM-based training, Tv decreased significantly and remained within lung-protective limits (4–6 ml/kg) across all subsequent sessions (*p* < 0.001). However, mask leakage consistently exceeded the 20% threshold, regardless of device or session.

**Discussion:**

RFM-based training significantly improved tidal volume control, supporting the adoption of lung-protective ventilation techniques among neonatology residents. Despite these gains, high levels of mask leakage persisted, suggesting the need for targeted instruction in mask handling and sealing. The retention of improved ventilation performance over three months highlights the educational value of RFM in neonatal resuscitation training.

## 1 Introduction

Each year in Brazil, approximately 500,000 newborns require assistance to initiate and sustain breathing at birth. Among them, a significant proportion of low-birthweight infants require manual ventilatory support to facilitate their adaptation to the extrauterine environment (1, 2).

Timely and effective resuscitation is crucial for reducing neonatal morbidity and mortality. Positive-pressure ventilation (PPV), which is initially delivered via a face mask, is a cornerstone of neonatal resuscitation, as it establishes functional residual capacity (FRC), delivers an adequate tidal volume (Tv), and stimulates spontaneous breathing (3–5).

Manual ventilation can be performed using devices such as a self-inflating bag (SIB) or a T-piece resuscitator. Compared with the SIB, the T-piece resuscitator provides superior control of peak inspiratory pressure (PIP) and positive end-expiratory pressure (PEEP), which ensures more consistent ventilation (6).

Despite its critical role, manual ventilation is inherently challenging. Chest expansion is an unreliable indicator of effective pulmonary ventilation and does not accurately reflect tidal volume delivery or the extent of air leakage. This limitation increases the risk of lung injury (3) and compromises resuscitation outcomes. Furthermore, operators frequently underestimate mask leakage during PPV regardless of their level of experience or familiarity with the equipment (4, 7). Respiratory function monitoring (RFM) is a tool that detects and corrects important ventilatory parameters in real time, such as tidal volume and pressure delivery, as well as leaks around the face mask, thereby optimizing ventilation parameters and improving neonatal outcomes (7, 8).

Previous studies have shown that RFM is an effective training tool for manual ventilation and aids clinicians in refining facial mask positioning to minimize leakage (7, 9). Notably, the skills acquired through RFM training have been shown to persist for at least one month post-training (9).

To enhance neonatology education, residency training should integrate both up-to-date theoretical instruction and the hands-on simulation of real-life clinical scenarios. The mastery of manual ventilation is a fundamental competency for neonatology residents and improving training strategies is critical for ensuring optimal neonatal care.

The aim of this study is to evaluate whether RFM increases tidal volume delivery and reduces leakage during manual ventilation training among neonatology residents.

## 2 Materials and methods

### 2.1 Study design and participants

We conducted a prospective experimental study using a full-body newborn baby manikin (140 RN, Simulacare, São Paulo—http://www.simulacare.com.br/140RN-Manequim-Bebe-Para-RCP.asp) that was fully articulated at the limbs and head to permit realistic positioning. The chest of the manikin also features high anatomical realism and is covered with skin-like material that closely mimics a real baby's appearance.

Twenty-three (23) neonatology residents from the following four distinct neonatal residency programs participated in this study: Hospital Israelita Albert Einstein, Faculty of Medical Sciences of Santa Casa (Faculdade de Ciências Médicas da Santa Casa de São Paulo), Botucatu Medical School, São Paulo State University (UNESP), and the Vila Nova Cachoeinha Hospital and Maternity (Hospital e Maternidade Vila Nova Cachoeirinha).

### 2.2 Ethical considerations

This study was approved by the Research Ethics Committees of all the participating institutions (CAAE: 64371517.7.0000.0071). All the residents provided written informed consent before participation. Confidentiality was maintained by anonymizing the data to ensured that no results were linked to individual participants. There are no conflicts of interest associated with this study.

### 2.3 Study protocol

**Figure 1:**
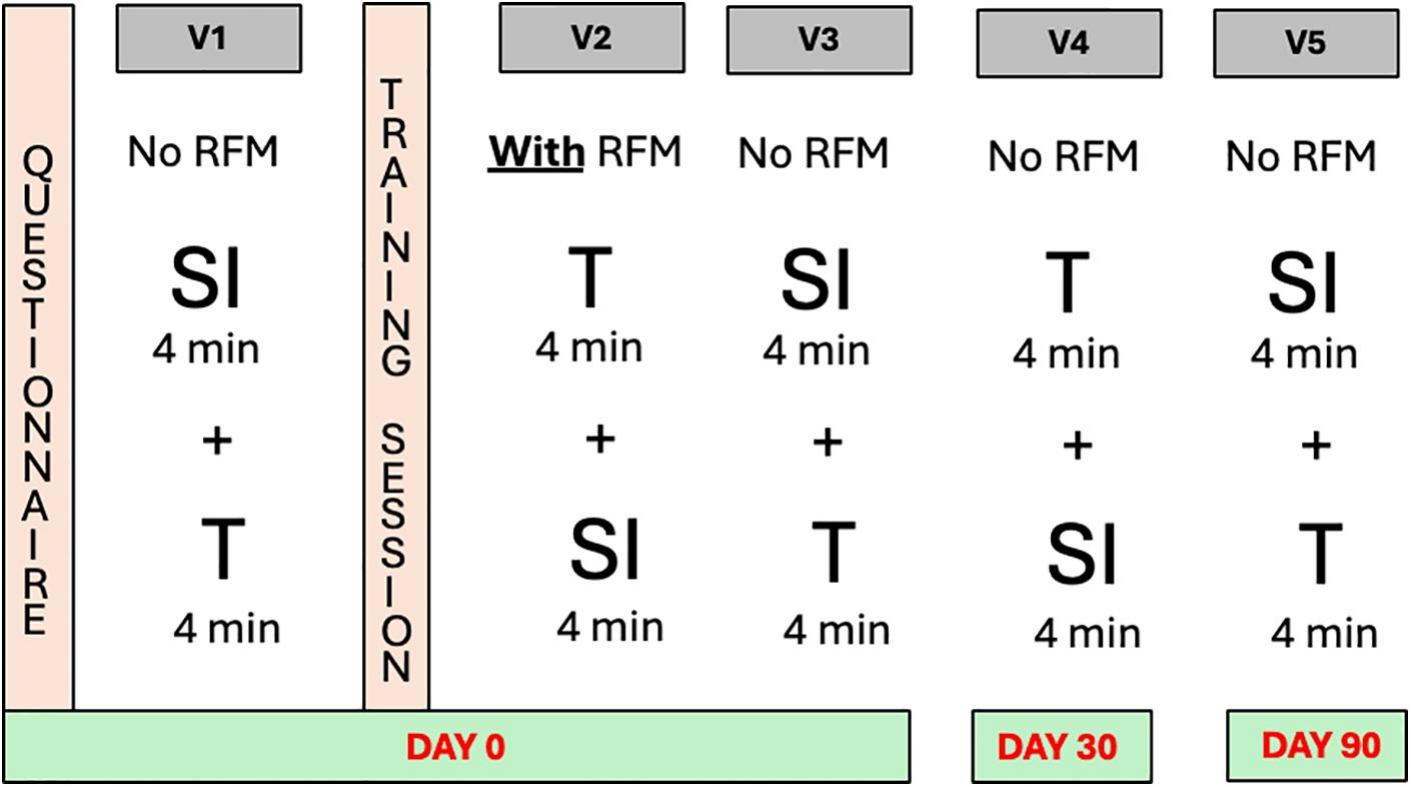
Breakdown of the different training days and their respective activities. QUESTIONAIRE: Before the first ventilation each resident answered a questionnaire regarding personal and demographic information; Vl: First ventilation—without RFM data; TRAINNING: 15 minute theoretical class covering the most relevant data on the RFM; V2: second ventilation—with RFM data; V3: third ventilation—without RFM data; V4: fourth ventilation—without RFM (performed one month after DAY O); V5: fifth ventilation without RFM data (performed three months after DAY O); RFM: respiratory function monitor; SI: self-inflating bag; T: T-piece resuscitator.

Data were collected at the three following time points: baseline (Day 0), one month (Day 30), and three months (Day 90). The methodology was standardized across all participating centers. The first study session consisted of five sequential activities (Figure 1).

### 2.4 Ventilation training sessions

Before the first ventilation session, participants completed a questionnaire detailing their personal and demographic information (Table 1). Then, they were presented with a standardized neonatal resuscitation scenario involving a full-term neonatal manikin simulating an apneic newborn with an estimated weight of 2.5 kg and no esophageal replica, experiencing apnea, with bradycardia (<100 bpm), and requiring positive pressure ventilation (PPV) as per the national neonatal resuscitation guidelines—Brazilian Society of Pediatrics Guidelines on Neonatal Resuscitation (2022 update) (1).

**Table 1:** Demographic characteristics of the 23 participating residents.

Demographic data	Results
Age [Mean (min—max)]	29 (24–35) years
Sex *N* (%)	
Female	21 (91.3)
Male	2 (8.7)
Institution of residency *N* (%)	
Universidade Estadual Paulista—Botucatu	8 (34.8)
Hospital e Maternidade Vila Nova Cachoeirinha	7 (30.4)
Faculdade de Ciências Médicas Santa Casa de São Paulo	5 (21.7)
Hospital Israelita Albert Einstein	3 (13.1)
Neonatal residency year *N* (%)	
First	14 (60.9)
Second	9 (39.1)
Courses taken *N* (%)	
Neonatal resuscitation	23 (100)
Resuscitation of preterm below 34 weeks gestation	8 (34.8)
High risk newborn transport	8 (34.8)
Previous equipment use *N* (%)	
Self-inflating bag	23 (100)
T-piece resuscitator	23 (100)
Respiratory function monitor	3 (13)

Participants performed manual ventilation using typical techniques without access to ventilatory monitoring data and relying solely on chest expansion for guidance (V1). Each resident completed two four-minute ventilation sessions using both a self-inflating bag (SIB—Hudson RCI) without an inbuilt manometer and a T-piece resuscitator (BabyPuff®, Fanem LTDA, Brazil), with the order of device use randomized via opaque envelope selection. Residents were free to determine inspiratory and expiratory pressures for both types of equipment at their discretion throughout all ventilation sessions (V1–V5).

Following V1, residents received a 15 minute standardized theoretical training session on respiratory monitoring, which was delivered by the same instructor. The session emphasized the importance of maintaining tidal volume (Tv) within the lung-protective range of 4–6 ml/kg, the benefits of using a PEEP valve with the SIB to preserve functional residual capacity (FRC), and the interpretation of flow-time curves to diagnose and correct leaks through proper positioning of the face mask. Participants also practiced ventilation using the RFM and were trained to identify and interpret key data displayed on the monitor.

Immediately after training, participants completed a second ventilation session (V2) with access to the RFM data and using the opposite device order from V1. A third ventilation session (V3) immediately followed, again without RFM data, but the device order was reversed from V2. Learning retention was assessed after one month (V4) and three months (V5), with participants performing ventilation using both devices without access to RFM data.

### 2.5 Respiratory monitoring and data acquisition

Throughout the ventilation sessions (V1–V5), the manikin was connected to a computerized data acquisition system that recorded the ventilatory pressure via a pressure sensor (Validyne, model DP45-24) and the inspiratory and expiratory flow via a pneumotachograph (Hans Rudolph Inc., Kansas City, USA). After each dataset collection session, devices were calibrated using an external syringe to ensure the integrity of the manikin's lungs and to verify the proper function of the pressure and flow sensors. These sensors were connected to a continuous data acquisition system (LabVIEW 5.1, National Instruments), which was specifically developed for this study by R. A. Eletro Sistemas LTDA (Campinas, Brazil).

**Figure 2:**
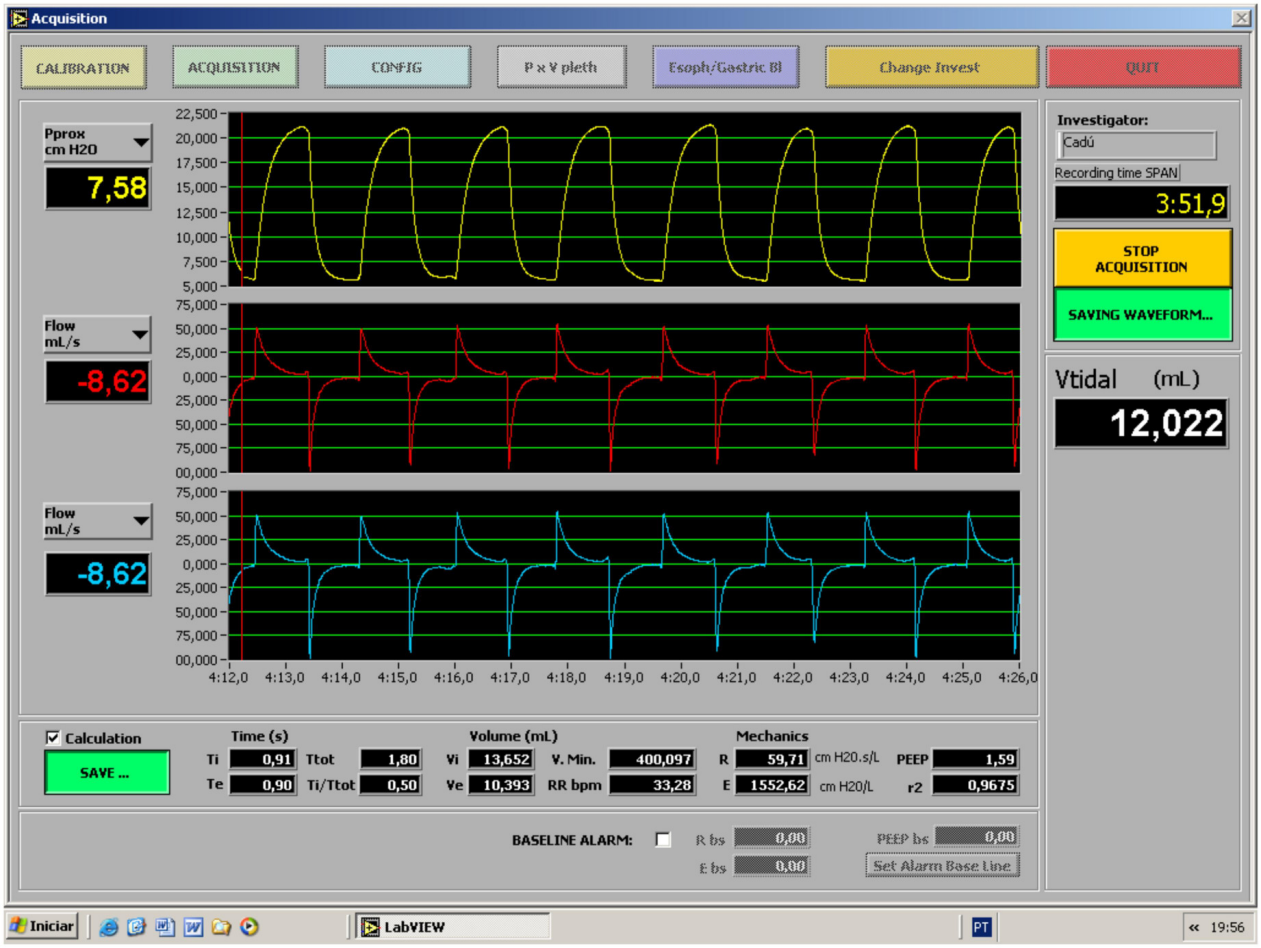
The layout of the RFM and highlighting the recorded ventilation parameters.

The Figure 2 illustrates the RFM.

**Figure 3:**
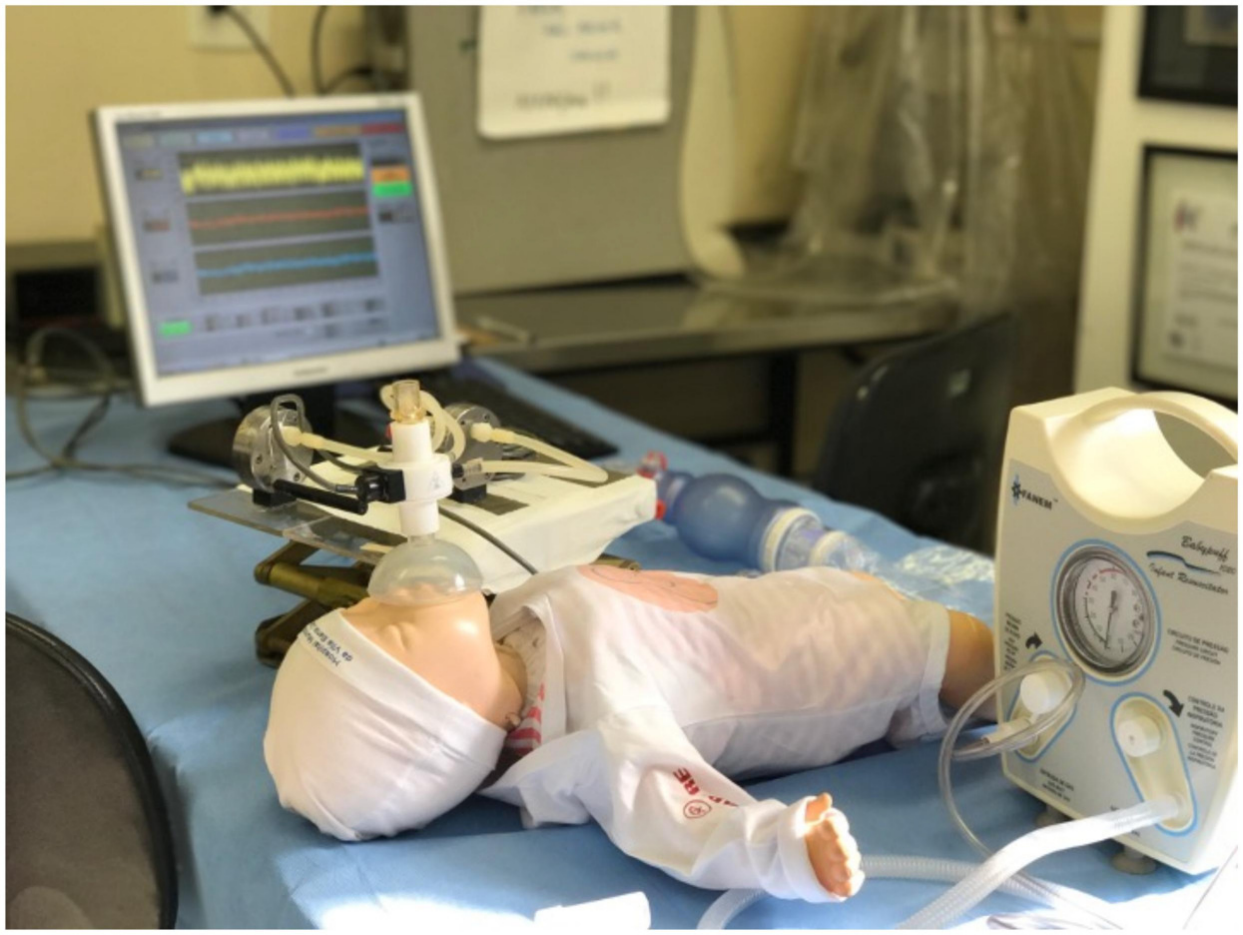
Layout of the study as the neonatal manikin's ventilation is assessed by the respiratory function monitor attached to the face mask.

The study setup is displayed in Figure 3 and ventilation of the neonatal manikin was monitored in real time using an RFM connected to the face mask, which captures airflow and pressure data during resuscitation efforts.

Tidal volume (Tv) was calculated by integrating flow data over time using a pneumotachograph. Facial mask leakage was determined by measuring the volume of air that failed to return from the manikin lung to the flow sensor during expiration and used the following formula:

$$\begin{array}{rl} \mathrm{Leakage}\;(\text{\%}) & =[(\mathrm{Inspiratory}\;\mathrm{Tv}-\mathrm{Expiratory}\;\mathrm{Tv})\times 100]\\ & \;/\mathrm{Inspiratory}\;\mathrm{Tv} \end{array}$$

### 2.6 Statistical analysis

A sample size of 23 residents allowed for a paired *t* test analysis to detect a 17% difference in ventilation performance with and without RFM data when the T-piece resuscitator was used and a 12.5% difference when the SIB was used. Power was set at 80% with a 0.05 level of significance.

A total of 23 residents were chosen on the basis of convenience and this also reflects the typical group size in training sessions conducted by the Neonatal Resuscitation Program of the Brazilian Society of Pediatrics. Moreover, this sample size permitted the use of a paired test to detect a 17% difference between ventilations without and with the RFM using the T-piece and a 12.5% difference when the self-inflating bag was used and assuming a statistical power of 80% and a significance level of 0.05. Overall, data followed a normal distribution.

Ventilation data (V1–V5) are presented as the means, standard deviations, medians, and ranges. Long-term effects of the RFM were assessed using mixed linear models, which accounted for repeated measures within participants and variations across device conditions. The model adequacy was assessed using residual diagnostics. All statistical analyses were performed using SPSS (version 3) with a significance threshold of *p* < 0.05.

## 3 Results

Twenty-three neonatal residents were included in the first phase of the study and their demographic data are presented in Table 1. All residents attended sessions V1–V3; however, six were lost in V4 and five in V5 because of vacations or international internships. Linear mixed model fitting was employed to account for missing data and to preserve the statistical validity of the analysis.

**Figure 4:**
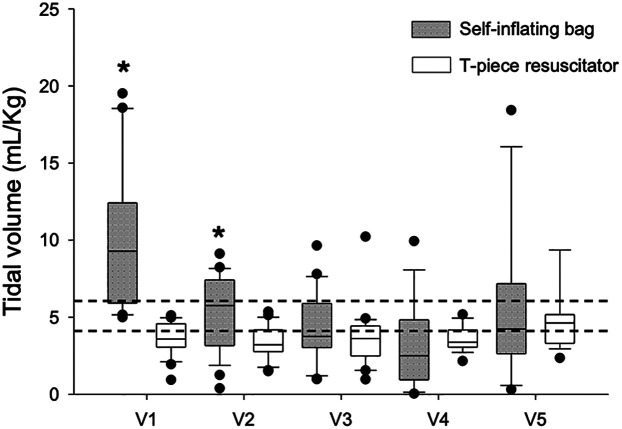
Expiratory tidal volume and type of equipment used for manual ventilation. Box plots show median, interquartile interval and 5th and 95th percentile whiskers. Dots are outliers. Dotted lines are limits for expiratory Tv within the lung protective range of 4–6 mL/Ukg. *P*­values were corrected by the Bonferroni method for multiple comparisons. *p < 0.05 vs. SIB and T-piece resuscitator.

An expiratory tidal volume (Vt) between 4 and 6 ml/kg was considered lung protective in accordance with Brazilian ventilatory guidelines. The mean expiratory Tv/kg for the SIB decreased after monitor training from 10.43 ml/kg (9.15–11.72) in V1 to 5.41 ml/kg (4.13–6.70) in V2 (*p* = 0.016), whereas it remained stable and within the protective parameters for the T-piece resuscitator before training [3.82 ml/kg (2.53–5.10) in V1 to 5.75 ml/kg (4.3–7.18) in V5; *p* = 0.164]. Thus, when the SIB was used, residents delivered excessive Tv in V1 compared to the T-piece [10.43 ml/kg (9.15–11.72) vs. 3.82 ml/kg (2.53–5.10) *p* < 0.001, respectively], and they were able to deliver ventilation within the protective range in all subsequent ventilation sessions after monitoring training (V3–V5) with the SIB and obtained exp Tv/kg comparable to the T-piece (3.37–5.95 ml/kg; *p* = 0.22–0.95) (Figure 4).

**Figure 5:**
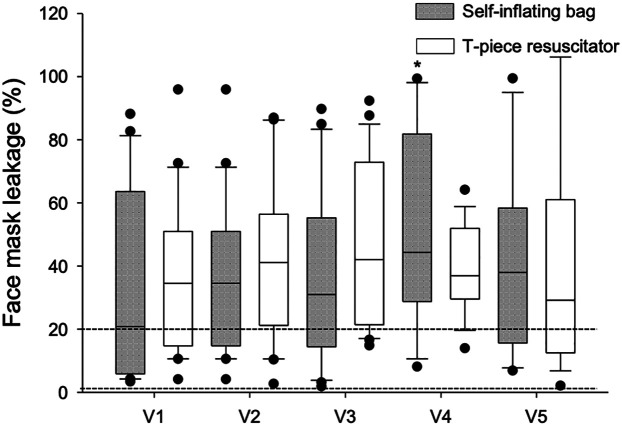
Mean face mask leakage values and type of equipment used for manual ventilation. Box plots show median, interquartile interval and 5th and 95th percentile whiskers. Dots are outliers. Dotted lines are acceptable leakage limits for manual ventilation. *P*-values were corrected by the Bonferroni method for multiple comparisons. *p < 0.05 vs. SIB and T-piece resuscitator.

The leakage remained above the 20% target (represented by horizontal lines in Figure 5) in all ventilations and regardless of the type of equipment. The mean facial mask leakage for SIBs ranged from 31.7% (19.99–43.47) in V1 to 50.1% (36.55–63.61) in V4, which was statistically significant (*p* = 0.047). When the T-piece was used it ranged from 36% (24.26–47.74) in V1 to 47.8% (36.07–59.55) in V3, but the difference was not statistically significant (*p* = 0.585) as shown in Figure 5.

## 4 Discussion

This study demonstrated that respiratory function monitoring (RFM) is a valuable tool for training neonatology residents in proper manual ventilation techniques. Specifically, this training aids in reducing the tidal volume (Tv) delivered during manual ventilation with self-inflating bags (SIBs). Training neonatology residents is critical given the importance and complexity of manual ventilation in the delivery room. Our findings indicate that a single RFM training session resulted in learning retention for up to three months. To our knowledge, this is the first study to focus solely on training neonatology residents in manual ventilation using an RFM while also evaluating long-term learning retention beyond one month.

Ericsson's concept of deliberate practice for medical skills training emphasizes well-defined learning objectives, precise performance measurements, structured repetition, and constructive feedback (10). Our study incorporated these principles by setting clear training goals, measuring performance objectively, and providing residents with real-time feedback on their ventilation technique.

Following theoretical training, residents understood that their primary objective was to apply a lung-protective manual ventilation strategy. This strategy involved optimizing the tidal volume within the 4–6 ml/kg range and minimizing leaks below 20%. The hands-on experience with the RFM (V2) reinforced these targets by providing continuous pulmonary mechanics data. Each resident completed ten four-minute ventilation sessions, including follow-up evaluations at one and three months, and they received feedback on their performance.

Prior to training (V1), the average expiratory Tv with SIBs was 10.43 ml/kg, which was well above the protective range. However, immediately after training (V2), the Tv values rapidly fell within the target range and remained stable throughout subsequent sessions (V3–V5) and aligning with those of the T-piece resuscitator. These results are comparable to those reported by Kelm et al., who reported a significant reduction in Tv after RFM training, with initial excessive tidal volumes decreasing and stabilizing within the protective range (11). However, unlike our study, Kelm et al. did not evaluate learning retention beyond one month and they did not detail participants' levels of experience or the specifics of their RFM training protocol.

Similarly, O'Currain et al. reported excessive Tv delivery among 388 health care professionals receiving neonatal resuscitation training (12). Their study revealed that access to RFM data led to increased tidal volumes by reducing facial mask leaks. This highlights the significant impact of leakage on Tv delivery. In contrast, our study revealed that residents did not effectively recognize or reduce leaks, which remained excessive across all the ventilation sessions (V1–V5) and could potentially lead to ineffective resuscitation in real-life scenarios. Despite the reduction in expiratory Tv following RFM training, the failure to address mask leakage suggests that our training approach did not adequately teach residents to interpret the flow/time curve for leak correction. We believe that a simplified RFM layout that displays mask leakage as a percentage, rather than through flow–time curves, facilitates interpretation and permits quicker adjustment of the mask technique. This approach has also been supported by recent studies. In a crossover trial, Chathasaigh et al. (13) compared different RFM interface designs and reported significantly lower mask leakage when data were presented as a percentage; the median leakage was 11% (7%–26%) among 51 participants. Similarly, a large multicenter trial conducted by Dalley et al. (14) evaluated 50 clinicians that were trained using the Life Box RFM and reported leakage as a percentage. Their findings revealed median leak values of 11% (3%–22%) during initial training and 16% (6%–26%) at the three-month follow-up. Notably, in both sessions, leaks exceeding 60% were observed in only 5% of all inflations. These findings are consistent with those of Dvorsky et al. (15), who reported significantly lower leakage in the “full-access group,” where both the supervisor and the participant could view the feedback monitor. In that group, medical students achieved a mean leak rate of 34.9%. A key factor in our study was the design of the RFM display, which appeared cluttered and difficult for residents to interpret. While the numerical Tv values were prominently displayed, other critical parameters, such as the pressure/time and flow/time curves, were less visible.

Previous research by Takatori et al. (16) suggested that simplifying monitor displays, such as incorporating numerical leak percentages and visual indicators (e.g., color-coded happy or sad faces), can enhance usability and improve ventilation technique adjustments in real time. Future studies should explore whether optimized display designs lead to better learning outcomes.

A notable limitation of our study is the use of a neonatal manikins, which do not replicate the dynamic changes in lung compliance seen in real newborns. This may explain the stable ventilation results observed with the T-piece resuscitator. Additionally, our samples were selected on the basis of convenience, although the demographic data indicate adequate representation.

In conclusion, RFM-based training effectively enhances manual ventilation techniques among neonatology residents by minimizing excessive expiratory tidal volumes when self-inflating bags are used. However, this outcome did not lead to a reduction in facial mask leakage, which highlights the need for improved training strategies in leak recognition and correction. We believe that residents find curve interpretation challenging; therefore, we recommend a monitor layout that is cleaner and more focused on numerical data than graphs, as this facilitates understanding and enables quicker adjustments to the ventilation technique. Furthermore, training benefits persisted for at least three months, as indicated by the sustained Tv values occurring within the protective range. Future research should focus on refining RFM display designs to enhance the user experience and facilitate more precise manual ventilation techniques.

## Data Availability

The original contributions presented in the study are included in the article/Supplementary Material, further inquiries can be directed to the corresponding author.
